# Patient perspective on living with mild hemophilia in Germany: results from a nationwide survey

**DOI:** 10.3389/fmed.2024.1347024

**Published:** 2024-02-05

**Authors:** Rosa Sonja Alesci, Georg Goldmann, Susan Halimeh, Katharina Holstein, Christoph Königs, Wolfgang Miesbach, Christian Pfrepper, Martin Olivieri

**Affiliations:** ^1^IMD Blood Coagulation Center Hochtaunus, Bad Homburg, Germany; ^2^Institute of Experimental Hematology and Transfusion Medicine, University of Bonn, Bonn, Germany; ^3^Blood Coagulation Center Rhein-Ruhr, Duisburg-Altstadt, Germany; ^4^II. Medical Department, Coagulation and Hemophilia Center, University Medical Center Hamburg-Eppendorf, Hamburg, Germany; ^5^Department of Paediatrics and Adolescent Medicine, Clinical and Molecular Haemostasis, Goethe University, University Hospital Frankfurt, Frankfurt, Germany; ^6^Department of Hemostaseology and Hemophilia Center, Medical Clinic 2, University Hospital Frankfurt, Frankfurt, Germany; ^7^Division of Hemostaseology, Department of Hematology, Cellular Therapy, Hemostaseology and Infectiology, University of Leipzig Medical Center, Leipzig, Germany; ^8^Pediatric Thrombosis and Hemostasis Unit, Pediatric Hemophilia Center, Dr. von Hauner Children’s Hospital, LMU München, Munich, Germany

**Keywords:** mild hemophilia, hemophilia A, hemophilia B, care reality, joint bleeding, quality of life

## Abstract

**Introduction:**

The disease burden and bleeding risk of patients with mild hemophilia may be underestimated. Their health-related quality of life (QoL) may be negatively impacted by insufficient treatment and bleed-related joint damage connected to a potentially delayed diagnosis.

**Aim:**

This study aims to gain information on the care reality and QoL of patients aged ≥12 years with mild hemophilia in Germany.

**Methods:**

An anonymous cross-sectional patient survey using standardized questionnaires was conducted in a validated electronic patient-reported outcome system. Medical specialists, hemophilia centers, patient organizations, and support groups across Germany invited the patients.

**Results:**

A total of 43 patients (35 patients with hemophilia A, 5 patients with hemophilia B, and 3 patients for whom the information was missing) with a median age of 33 years were analyzed. The median age at diagnosis was 6.0 years (interquartile range [IQR] 2.0–15.0), and the median factor activity was 14.0% (IQR 12.0–25.0). Nearly 85% of the patients received factor concentrates in the past, and the most common reasons for the treatment were surgery or joint bleeding (each 65.6%). Half of the patients who provided feedback experienced complications during bleeding episodes. Prophylactic treatment with factor concentrates was rare (10.3%). The patients had minor problems regarding their health status.

**Conclusion:**

Bleeding complications and joint bleeding, in particular, may be highly underestimated in patients with mild hemophilia, highlighting a medical need in this population. Patients with a potential benefit from prophylaxis need to be identified. Mild hemophilia has a negative impact on patients’ QoL. Hemophilia centers satisfied the patients’ needs. Further research is needed to address the current lack of awareness and improve adequate treatment in the future.

## 1 Introduction

Hemophilia refers to a rare congenital bleeding disorder (1) mainly affecting men (2). It is characterized by a coagulation factor deficiency of factor VIII (FVIII; hemophilia A, HA) or factor IX (FIX; hemophilia B, HB) (1).

Normal factor (FVIII or FIX) activity ranges from 50 to 150% (3) Residual factor activity in a patient’s plasma is used to differentiate three severity degrees of hemophilia: mild with a relatively broad range of >5 to <40%, moderate with 1 to 5%, and severe with <1% of normal factor activity. Those with the normal factor activity ranging from 40 to 50% are not yet classified (4) Mild hemophilia with its wide range shows a varying phenotype. Some patients may present with a bleeding phenotype overlapping with severe hemophilia, possibly requiring prophylactic treatment (5), highlighting the importance of an individualized treatment strategy (1, 6).

Unlike severe hemophilia, mild phenotypes are often diagnosed later in life (5, 7), usually as a result of extended bleeding episodes provoked by injury or medical interventions; however, recurrent spontaneous bleeding episodes are rare in mild hemophilia. Recently, patient age was reported to correlate with arthropathy, a most relevant factor negatively affecting the quality of life (QoL) (7).

In mild HA, the development of FVIII neutralizing antibodies (inhibitors) is usually due to intensive exposure to factor concentrates (7, 8); the risk of inhibitor development may be associated with certain F8 mutations (8). While occurring less frequently in patients with mild or moderate HA compared to the severe form, these conditions pose a lifelong risk (8). Inhibitors can increase the severity of bleeding episodes (7) and may even shift a mild phenotype to a severe one (9), thereby complicating the treatment (10). FIX inhibitors are rare (1.5 to 3% of patients) and are almost exclusively found in patients with severe HB (1, 11), for whom an HB-specific formal clinical guidance may be lacking due the rarity of their condition (12). FIX inhibitor formation is unrelated to the type of FIX clotting factor concentrate (CFC) and is considered a most serious complication as anaphylaxis and nephrotic syndrome may occur (11).

Exact numbers of patients with (mild) hemophilia are unknown (1) due to various reasons (2, 7), and the number of undiagnosed patients is likely to far exceed (1) than those reported (2): in 2020, 4,518 patients with HA (mild type: 738 patients) and 860 patients with HB (mild type: 152 patients) were newly registered in the German Hemophilia Registry (13).

In patients with mild HA without medical contraindications, desmopressin is recommended for minor bleeding episodes, surgeries, and other invasive procedures (1). Desmopressin, a synthetic vasopressin analog, induces von Willebrand factor release from endothelial organelles and simultaneously increases FVIII levels by two- to six-fold (7, 14, 15). Factor replacement therapy may be required upon serious trauma or surgical procedures. Anti-fibrinolytic therapy with or without desmopressin can be used for the treatment of mucosal bleeding or invasive dental procedures. The use of desmopressin is cheaper than CFCs, avoids exposure to FVIII concentrates, and reduces the risk of inhibitor development (1, 16). It is ineffective in patients with HB (1, 7).

Patients with mild hemophilia or their families may initially lack awareness of the disease (9), leading to a delay in diagnosis (7). Suspicion may only be raised upon severe symptom development or complications, such as prolonged bleeding episodes after medical interventions, resulting from inadequate management. The literature on mild hemophilia including diagnosis and management is limited (7), and mild hemophilia may be underdiagnosed and undertreated compared to severe hemophilia (7, 17).

The current treatment of severe hemophilia aims to change its phenotype to that of moderate-to-mild hemophilia (1, 18) by prophylaxis (1). However, for patients with mild-to-moderate hemophilia, unmet needs (18), such as recommendations for physical activity (19), remain.

This survey aimed to investigate the care reality and QoL of patients with mild hemophilia, as daily problems and restraints may remain largely neglected (7).

## 2 Materials and methods

This cross-sectional survey was conducted to collect information on the care reality and the QoL of patients aged ≥12 years in Germany, diagnosed with mild HA or HB. The main objectives of this study were to assess the patient satisfaction with the therapy and support provided by hemophilia centers, the impact of hemophilia on daily life, QoL [EQ-5D-5L questionnaire (20)], and medical treatments. The web-based survey collected anonymous data directly from patients in a single session using standardized electronic questionnaires filled out in the AMS-ePRO^®^ tool, a validated electronic patient-reported outcome system. Consequently, the captured data were encrypted.

Various medical specialists, hemophilia centers, patient organizations, and support groups across Germany distributed invitations. Distribution and individualized one-time QR codes on invitations were not tracked, thereby ensuring anonymity. To minimize the selection bias, invitations were handed out to all eligible patients. Patients accessed the survey using their own smartphones/tablets by scanning the QR code. Internet connection via a standard web browser was secured by hypertext transfer protocol secure, and the database server was hosted under controlled conditions in an off-site facility.

Easy-to-complete lay language questions focused on demographics, diagnosis, and main objectives discussed earlier. It is important to note that drug-related adverse events were not collected, and there were no free-text fields. However, respondents were able to skip questions, and while logged in, questions could be answered in any order and ticked answers could be amended. Data capture could end prematurely, and re-entry was not possible. Logic and plausibility checks were implemented to ensure data quality and to minimize data inconsistencies.

Based on the number of patients and demographics in Germany, 150–200 patients were expected to participate. The sample size was based on the estimated number of suitable patients and was not formally calculated, as this was an exploratory survey without formal hypothesis testing. Statistical analyses were conducted using SAS^®^ (v9.4 or later; SAS Institute Inc., Cary, NC, United States). The analysis set included all eligible patients who answered at least one question. All results were reported descriptively. Continuous variables were represented using mean, standard deviation, median, quartiles (Q1 and Q3), range (minimum/maximum), and the number of missing values. Categorical variables were represented as absolute and percentage frequencies of answers. All available data were included in the analyses and summarized as far as possible. Unless otherwise specified, missing data were not replaced. The survey started in September 2021 and ended in July 2022.

## 3 Results

### 3.1 Patient characteristics

A total of 44 datasets were collected, and 43 were included in the final analysis set; one was excluded because it was a test input. The median age was 33.0 years (min–max 12–75; 41 answers; Table 1). The sex of the patients was recorded. The median age at the initial diagnosis of mild hemophilia was 6.0 years (min–max 0–37; 39 answers). Most patients had HA (87.5%, 40 answers).

**Table 1:** Self-reported patient characteristics (full analysis set: *N* = 43).

Patient characteristics	FAS *N* = 43
*Age (years)* ^a^	
Median (IQR)	33.0 (23.0–48.0)
Mean (SD)	36.0 (16.8)
Min–Max	12–75
*Age at diagnosis of mild hemophilia (years)* ^b^	
Median (IQR)	6.0 (2.0–15.0)
Mean (SD)	10.6 (10.6)
Min–Max	0–37
Unknown, *n* (%)^†^	1 (2.4)
*Hemophilia type, n (%)* ^c^	
Mild hemophilia A	35 (87.5)
Mild hemophilia B	5 (12.5)
*Factor activity at diagnosis* ^d^	
Median (IQR)	14.0 (12.0–25.0)
Min–Max	4–55
Unknown, *n* (%)^†^	10 (23.8)
*Reason for which blood coagulation was checked at hemophilia diagnosis, n (%)* ^‡^ ^,^^e^	
Familial predisposition	19 (46.3)
Bleeding episode during/after surgery or dental treatment	15 (36.6)
Increased hematoma frequency/intensity	10 (24.4)
Problems with stopping the bleeding episode	7 (17.1)
Coincidence	4 (9.8)
Frequent and/or severe nosebleeds	4 (9.8)
Unusual blood test results, indicating a possible coagulopathy	3 (7.3)
Frequent and/or severe gum bleeding	2 (4.9)
Unusual bleeding episode, e.g., knee, gastrointestinal	1 (2.4)
Unknown^†^	0 (0)
*Time elapsed between first symptoms and diagnosis of hemophilia, n (%)* ^f^	
<3 months	13 (33.3)
3 to 12 months	2 (5.1)
>1 year	10 (25.6)
Unknown^†^	14 (35.9)

Percentages are calculated for patients with non-missing data; number (missing) *n* = ^a^41 (2), ^b^39 (4), ^c^40 (3), ^d^30 (13), ^e^41 (2), ^f^39 (4).

† Patients could answer “I do not know”.

‡ Multiple answers were possible.

FAS, full analysis set; IQR, interquartile range 25–75; n, number; SD, standard deviation.

The median factor activity at diagnosis was 14.0% (min–max 4–55), with a mean (SD) activity of 18.1% (11.8), and 10 patients (23.8%) did not know their factor activity at diagnosis (30 answers). One patient with HA had factor levels within the lower limit of normal (4), experiencing increased hematoma frequency/intensity and unusual blood test results, suggesting a possible coagulopathy. In another patient with HA, factor levels below the limit of “mild” (4) were detected by coincidence.

Patients could provide multiple answers regarding the reasons for which blood coagulation was checked at hemophilia diagnosis (41 answers). The three most common reasons for hemophilia diagnosis were familial predisposition (46.3%), bleeding episodes during/after surgery or dental treatment (36.6%), and increased hematoma frequency or intensity (24.4%, 41 answers). The time elapsed between first symptoms and hemophilia diagnosis was <3 months for one-third of the patients (33.3%). Another third of the patients (35.9%) did not know how much time passed between first symptoms and hemophilia diagnosis. Approximately one-quarter of patients received their diagnosis >1 year after their first symptoms (39 answers, Table 1).

### 3.2 Previous hemophilia treatment

The majority of the patients (84.2%) had received the factor concentrate for hemophilia treatment in the past (38 answers, Table 2). The median age of first factor administration was 10.0 years (min–max 0–56; 27 answers; Table 2).

**Table 2:** Previous hemophilia treatment—type of treatment and age of first treatment administration (full analysis set: *N* = 43).

Characteristics	FAS *N* = 43
*Treatment with a factor concentrate in the past, n (%)* ^a^	
Yes	32 (84.2)
No	6 (15.8)
*Age at the first factor concentrate administration (years)* ^b^	
Median (IQR)	10.0 (6.0–19.0)
Mean (SD)	16.0 (14.1)
Min–Max	0–56
Unknown, *n* (%)^†^	5 (15.6)
*Treatment with desmopressin in the past, n (%)* ^c^	
Yes	11 (28.2)
No^‡^	20 (51.3)
Unknown^†^	8 (20.5)
*Age at the first desmopressin administration (years)* ^d^	
Median (IQR)	27.5 (9.0–29.0)
Mean (SD)	23.0 (14.9)
Min–Max	4–49
Unknown, *n* (%)^†^	3 (27.3)
*Complications due to untreated bleeding episodes, n (%)* ^e^	
Yes	19 (50.0)
No	18 (47.4)
Unknown^†^	1 (2.6)

Percentages are calculated for patients with non-missing data; number (missing) n = ^a^38 (5); ^b^27 (16); ^c^39 (4), ^d^8 (35), ^e^38 (5).

† Patients could answer “I do not know”.

‡ Includes 3 patients with hemophilia B; 17 patients (43.6%) with hemophilia A did not receive desmopressin.

FAS, full analysis set; IQR, interquartile range 25–75; n, number; SD, standard deviation.

Almost half of the patients with HA (43.6%) had not received desmopressin treatment in the past (39 answers). The median age at first desmopressin administration was 27.5 years (min–max 4–49); the mean age (SD) was 23.0 years (14.9, 8 answers, including three patients who could not recall).

Half of the patients (50.0%, 38 answers) experienced complications due to untreated bleeding episodes in the past.

### 3.3 Reasons for previous hemophilia treatment and for complications due to untreated bleeding episodes

Patients could provide multiple answers regarding the reasons for previous treatments (ranging from 11 to 32 answers). There were multiple reasons for the treatment with the factor concentrate (Table 3), with the most common being joint bleeding and/or a perioperative setting (21; 65.6% each). The most common reasons for using desmopressin were medical procedures (dental treatments and surgery) or accidents (11 answers). Spontaneous or joint bleeding was less frequently treated with desmopressin. The most common reasons for complications due to untreated bleeding episodes—19 patients (50%) who answered that they had experienced complications—were due to medical procedures, such as dental treatment (10/19 patients; 52.6%), surgery (11/19 patients; 57.9%), and joint bleeding (10/19 patients; 52/69%). Patients could provide multiple answers to this question.

**Table 3:** Reasons for previous hemophilia treatments and for complications due to untreated bleeding episodes (full analysis set: *N* = 43).

Characteristics	FAS *N* = 43
*Reason for treatment with factor concentrate, n (%)*^†^^,^^‡^^,^^a^	
Joint bleeding	21 (65.6)
Perioperative setting	21 (65.6)
Dental treatment/tooth replacement	19 (59.4)
Accident	15 (46.9)
Spontaneous bleeding	4 (12.5)
Unknown	0 (0)
*Reason for treatment with desmopressin, n (%)*^†^^,^ ^‡^^,^^b^	
Dental treatment/tooth replacement	6 (54.5)
Perioperative setting	4 (36.4)
Accident	3 (27.3)
Joint bleeding	2 (18.2)
Spontaneous bleeding	2 (18.2)
Unknown	0 (0)
*Reason for complications due to untreated bleeding episodes, n (%)*^†^^,^ ^‡^^,^^c^	
Bleeding after surgery	11 (57.9)
Dental treatment/tooth replacement	10 (52.6)
Joint bleeding	10 (52.6)
Accident	4 (21.1)
Spontaneous bleeding	2 (10.5)
Unknown	0 (0)

Percentages are calculated for patients with non-missing data; number (missing) n = ^a^32 (11), ^b^11 (32), ^c^19 (24).

† Patients could answer “I do not know”.

‡ Multiple answers were possible.

FAS, full analysis set; n, number.

### 3.4 Current hemophilia treatment and reasons for choosing prophylaxis with factor concentrate

Patients could provide multiple answers regarding their current treatment/the use of prophylaxis (39 answers). Most patients (69.2%) received the factor concentrate on-demand. One-quarter of the patients used medications other than factor concentrates, such as tranexamic acid or desmopressin. Furthermore, 59% of the patients received non-medicinal treatment or no medication for the treatment of bleeding episodes (Table 4) and used, e.g., plasters or dressings.

**Table 4:** Current hemophilia treatment of bleeding episodes and reasons for choosing prophylaxis with factor concentrate (full analysis set: *N* = 43).

Characteristics	FAS *N* = 43
*Current treatment/use of prophylaxis, n (%)*^†^^,^^a^	
Current treatment of bleeding episodes	
Factor concentrate if required	27 (69.2)
Non-medicinal treatment or no medication	23 (59.0)
Other medications except factor concentrate	10 (25.6)
*Current use of prophylaxis*	
Factor concentrate	4 (10.3)
*Use of factor concentrate with extended half-life, n (%)*^‡^^,^^b^	
No	8 (29.6)
Yes	2 (7.4)
Unknown	17 (63.0)
*Reasons for choosing prophylaxis with factor concentrate, n (%)*^†^^,^^c^	
Better bleeding episode control even in the case of non-apparent bleeding	4 (100.0)
Reduction of bleeding episode frequency	4 (100.0)
Reduction of bleeding episode severity	3 (75.0)
Desire for a more active life	2 (50.0)
Reduction of hospitalizations	2 (50.0)
Prevention of joint damage	1 (25.0)
Not specified	0 (0.0)

Percentages are calculated for patients with non-missing data; number (missing) n = ^a^39 (4), ^b^27 (16), ^c^4 (39).

† Multiple answers were possible.

‡ Based on patients using “prophylaxis” and/or “factor concentrate if required”.

FAS, full analysis set; n, number.

Additionally, four patients (10.3%), two with HA and two with HB, currently used prophylaxis with factor concentrate. They indicated multiple reasons for choosing prophylaxis (Table 4). The three most frequent reasons were a desire for better control of bleeding episodes even in the case of non-apparent bleeding (100%), a reduction of bleeding episode frequency (100%), or severity (75%). The desire for more safety in daily life or a more carefree life was not in focus (0%).

Concentrates with an extended half-life were rarely used in this patient population (27 answers; Table 4), and most patients (63%) were unaware of whether they were receiving them.

### 3.5 Visits at hemophilia centers

Patients visited hemophilia centers only when needed (55.3%) or on a regular basis, i.e., more often than every six months and every 6 or 12 months (44.7%; 38 answers). Half of those patients (nine, 52.9%) who needed regular visits (17 patients) attended the hemophilia center about every six months or only about once a year (seven, 41.2%). One patient required more frequent visits (Table 5).

**Table 5:** Frequency of visits to the hemophilia center, use of additional services, and patient diaries (full analysis set *N* = 43).

Characteristics	FAS *N* = 43
*Frequency of visits to the hemophilia center, n (%)* ^a^	
Only when needed	21 (55.3)
Regularly	17 (44.7)
*Visit frequency by patients who regularly visit the hemophilia center, n (%)* ^b^	
About every 6 months	9 (52.9)
About once a year	7 (41.2)
More often than every 6 months	1 (5.9)
*Use of additional services, n (%)*^†^^,^^c^	
No additional services	29 (74.4)
Online services of the IGH	6 (15.4)
Online services of the DHG	5 (12.8)
Online community	2 (5.1)
Personal exchange with other patients	2 (5.1)
*Tracking of bleeding episodes and treatments in a diary, n (%)* ^d^	
No	20 (54.1)
Yes	17 (45.9)
*Physician takes a diary into account when deciding on treatment, n (%)* ^e^	
No	6 (35.3)
Yes	11 (64.7)

Percentages are calculated for patients with non-missing data; number (missing) *n* = ^a^ 38 (5), ^b^ 17 (26), ^c^39 (4), ^d^37 (6), ^e^17 (26).

† Multiple answers were possible.

FAS, full analysis set; DHG: Deutsche Hämophiliegesellschaft e.V.; IGH: Interessengemeinschaft Hämophiler e.V.; n, number.

### 3.6 Satisfaction with treatment and support

**Figure 1:**
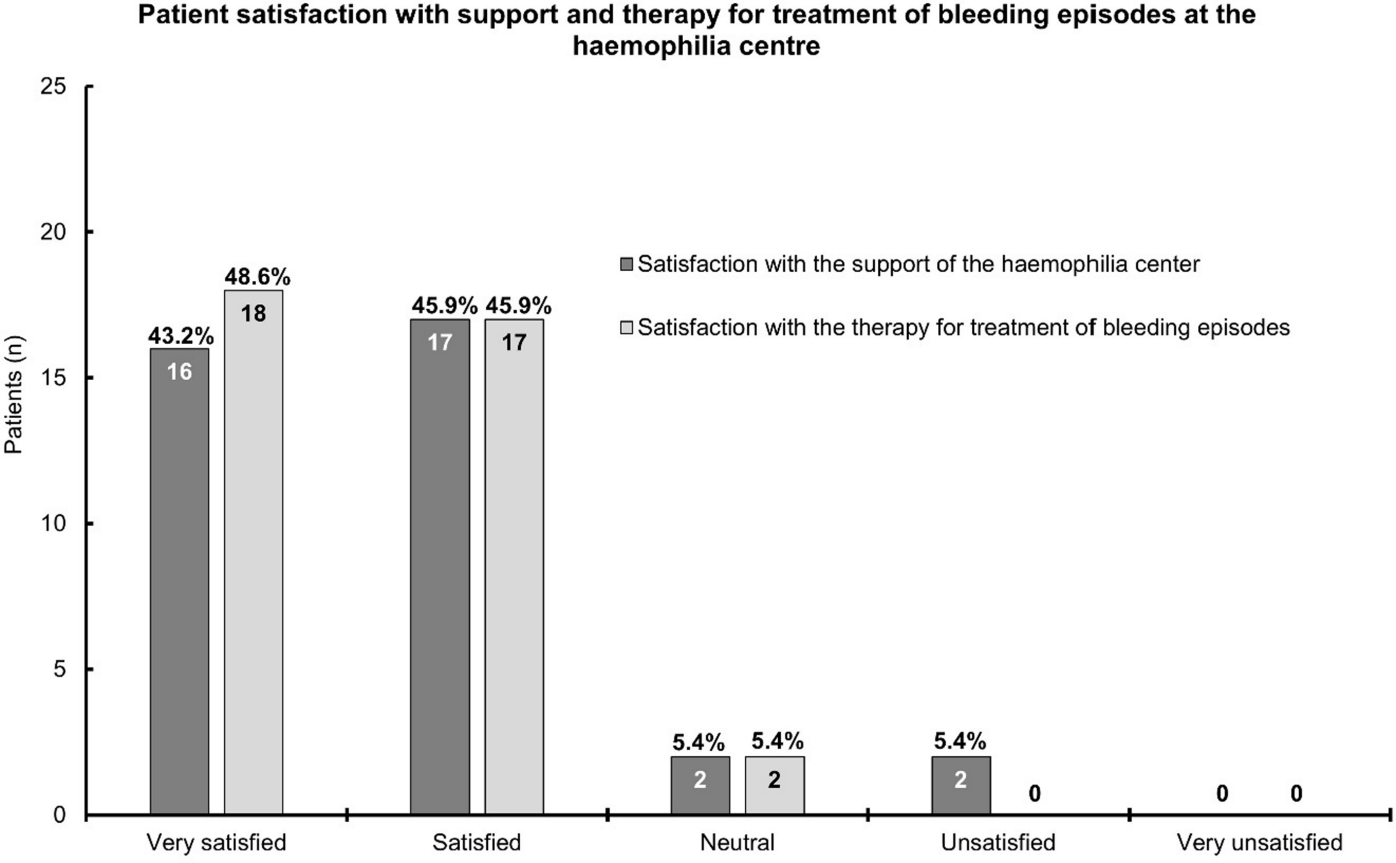
A bar chart depicting patient satisfaction with the support and therapy for the treatment of bleeding episodes at the hemophilia center. This chart is based on the information provided by 37 patients; the answers for six patients per topic were missing. The bars show the patient numbers at the inside end of the bars; the corresponding percentages are shown on top of the bars. Percentages relate to the number of patients who provided answers.

There were 37 patients who provided feedback on their satisfaction with the support and treatment received at the hemophilia center (Figure 1). Generally, patients were very satisfied (*n* = 16; 43.2%) or satisfied (n = 17; 45.9%) with the support from their hemophilia center. Then, two patients each (5.4%) were neutral or unsatisfied in this respect. The results were similar for the satisfaction with therapy for the treatment of bleeding episodes (very satisfied: *n* = 18; 48.6% and satisfied: *n* = 17; 45.9%). Two patients (5.4%) were neutral, and none was unsatisfied regarding the therapy for the treatment of bleeding episodes received at the hemophilia center. None of the patients was very unsatisfied with either the support or the treatment received at their hemophilia center (Figure 1).

The majority of the patients (73.7%) did not seek medical care from other physicians other than their hemophilia center, while approximately one-quarter of patients (26.3%) sought another medical opinion (38 answers).

Patients could provide multiple answers regarding the use of additional services for medical support (39 answers). Again, most patients (74.4%) did not utilize services in addition to their contact with the hemophilia centers (Table 5). Online services of patient societies or online communities/personal exchange with other patients were rarely used (39 answers). Nearly half of the patients (45.9%) used a diary to track bleeding episodes and treatments (37 answers; Table 5), while others did not. If patients used a diary, 64.7% of physicians incorporated this information into their decision-making (17 answers; Table 5).

### 3.7 Impact of hemophilia on daily life

**Figure 2:**
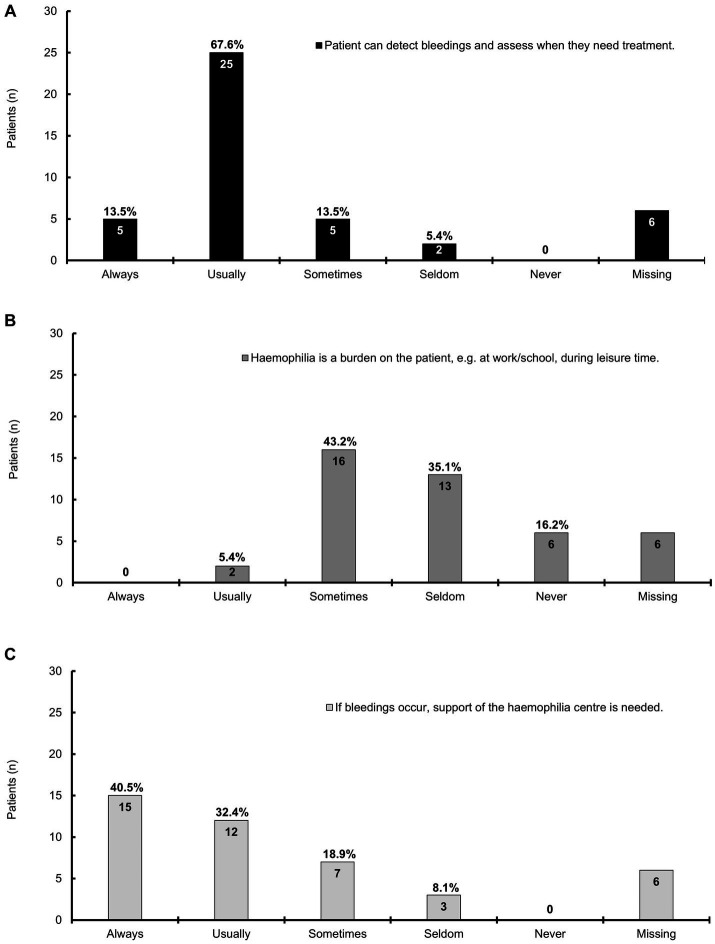
A bar chart representing the impact of mild hemophilia on daily life; this chart is based on the information provided by 37 patients; the answers for six patients were missing. The bars show the patient numbers at the inside end of the bars; the corresponding percentages are shown on top of the bars. Percentages relate to the number of patients who provided answers. **(A)** The majority of patients can always or usually detect bleeding episodes and assess when they need treatment; **(B)** Hemophilia is sometimes a burden on the patients, e.g., at work, school, or during leisure time; **(C)** The majority of patients always or usually needs support from the hemophilia center if bleeding occurs, which is sometimes the case for seven patients.

Answers were provided by 37 patients; most patients could usually (67.6%) or always (13.5%) detect the bleeding episodes and assess when they needed treatment (Figure 2A). Hemophilia was sometimes (43.2%) a burden, for example, at work, school, or during leisure time, and seldom for one-third (35.1%) of the patients (Figure 2B). Patients needed support always (40.5%), usually (32.4%), or sometimes (18.9%) from the hemophilia center upon bleeding episodes (Figure 2C).

### 3.8 Health questionnaire (EQ-5D-5L)

**Figure 3:**
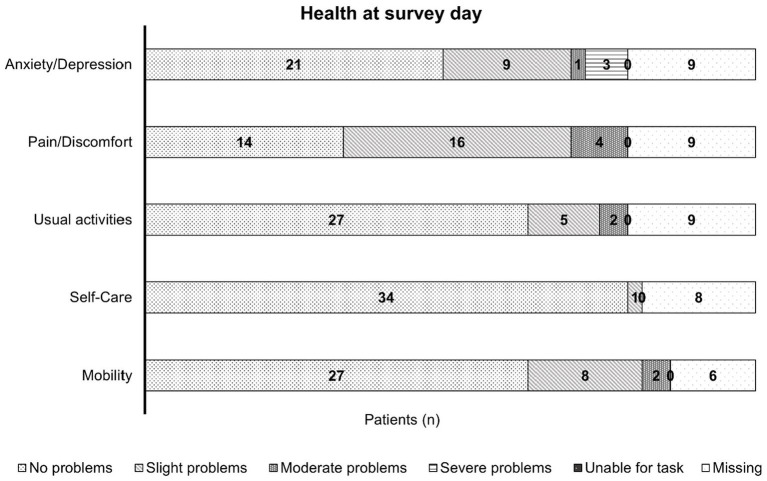
A bar chart representing the health of patients with mild hemophilia at the day of the survey; this chart is based on the information provided by 34, 35, and 37 patients, with missing information for six patients in mobility, eight patients in self-care, and nine patients each in their usual activities, pain/discomfort, and anxiety/depression. The bars show the patient numbers in the center of the respective bar section. Most patients experienced no limitations due to their condition in all five dimensions, except for the pain/discomfort dimension where nearly half of the patients experienced slight impairment.

EQ-5D-5L includes the dimensions “mobility”, “self-care”, “usual activities”, “pain/discomfort”, and “anxiety/depression”, with the ratings ranging from “no problems” to “unable” and “no” to “extreme” (20). The number of answers varied in different dimensions:

The answers were provided by 37 patients for mobility, 35 for self-care, and 34 patients for each of the dimensions: usual activities, pain/discomfort, and anxiety/depression. Most patients had no problems in all five dimensions, except for pain/discomfort (Figure 3). Here, the results were roughly similar for no and slight impairment (41.2 and 47.1%, respectively). For mobility and usual activities, >10% of the patients experienced slight problems (21.6 and 14.7%, respectively), and for anxiety/depression, >20% of the patients had slight symptoms (26.5%; Figure 3). Information was missing for eight patients regarding self-care and nine patients each for usual activities, pain/discomfort, and anxiety/depression.

The EQ-VAS scale allows patients to self-report their health status on a scale from 0 (“worst imaginable health”) to 100 (“best imaginable health”). The median EQ-VAS was 80.0 (range 30–100; based on 33 answers).

## 4 Discussion

This survey aimed to shed light on the reality of care for patients with mild hemophilia, as daily problems and restrains may remain largely neglected (7).

The proportion of hemophilia types in our patient population examined (HA: 87.5% and HB: 12.5%; 40 answers) was in line with that of the population affected (HA: 80 to 85% and HB: 15 to 20%) (1). The median age at diagnosis reported here (6.0 years) was similar to other publications (2.4 to 6.5 years), (9, 10, 21, 22), and the median factor activity at diagnosis (14%) was also in line with other findings (15%) (21). One patient indicated FVIII levels within the lower limit of normal (4), and medical history suggested a possible coagulopathy. Due to limited data, we can only speculate if this was due to temporarily elevated FVIII activity, or artifacts in applied assays (1), reporting an error or a misunderstanding by the patient. The two latter reasons or false low values (1) may be causal for the factor levels below the limit of “mild” (4), which were detected by coincidence in another patient with FVIII deficiency. Both patients were included in the analysis as local laboratory standards on categorization levels for hemophilia slightly differ.

The main reasons for diagnosis in this survey, such as familial predisposition, bleeding episodes during/after a surgery or dental treatment, and increased hematoma frequency or intensity, were also reported by other publications (7, 21). The diagnosis of mild hemophilia is often delayed compared to that of more severe phenotypes (7, 9, 18) and this delay is highly dependent on a country’s economic status (2, 9). Patients unaware of their condition may neglect symptoms until late (7, 9). The main reason for a late(r) diagnosis is the late(r) onset of bleeds as patients with a mild phenotype often do not have spontaneous bleeds and require “adequate” trauma (7).

In our survey, most patients (84.2%) had previously received the factor concentrate for the treatment of hemophilia. Other studies report lower proportions to treat bleedings (51 to 75%) (16, 21) or joint bleeds (40%) (21); an exact comparison of the results is limited as, due to limited data, we cannot report the exact specification of the CFC use. Generally, the use of CFCs bears the risk of inhibitor development, which may eventually complicate treatment (10, 23). The age at first FVIII treatment largely depends on the level of the baseline factor activity. In addition, patients with mild HA may receive their first CFC dose at 4.4 years. A known family history of HA may lead to an earlier treatment (3.9 years), while a negative family history can lead to a delay in therapy (6.4 years). Patients with mild HA and a negative familial history tended to be older if factor activities ranged between 10 and 15% (7.2 years) and 25 and 40% (12.1 years) (24). This is approximately in line with our cohort: The familial predisposition was known by less than half of the patients (46.3%), and they initiated the CFC treatment at a median age of 10.0 years, with a median factor activity at diagnosis of 14.0% (12.0–25.0%). The patients in our survey used factor concentrates mainly to treat joint bleeding and/or in a perioperative setting, as recommended (1).

Most patients (69.2%) received the factor concentrate on-demand as a current treatment option, which was lower than that reported in the DYNAMO study (98%), an international multicenter study including men aged 12 to 55 years with non-severe hemophilia (residual FVIII/IX activity: 2 to 35%) (21). Other patients (59.0%) chose non-medicinal treatment or no medication for the treatment of bleeding episodes and used plasters or dressings or other medications except the factor concentrate (25.6%; see below).

We observed a higher prophylaxis rate (10.3%) than the DYNAMO study (1.7% prophylaxis/intermittent prophylaxis), which included patients with a lower factor activity range (2 to 35%) determined by the central laboratory upon inclusion (21), while we report a patient-reported factor activity at diagnosis. The prophylaxis rate in our study was similar to the PROBE study (11.8% of men with mild hemophilia with regular/intermittent prophylaxis [*n* = 12] and 2.63% of women [*n* = 1] with regular prophylaxis) (25); however, we did not overtly offer the prophylaxis subcategories “regular/intermittent” (4) and did not ask for the participants’ sex. Knowledge on the benefits of prophylaxis is scarce (18). Patients with mild hemophilia may receive prophylaxis for the treatment of acute bleeding episodes or before invasive procedures (7, 25), potentially at a later stage than patients with severe hemophilia (5). There may be patients with mild hemophilia, who would benefit from prophylaxis, fostering “adequate” hemostasis and protection from the consequences of the diseases (5).

Factor concentrates with an extended half-life were rarely used by patients using “factor concentrate if required” and/or “on current prophylaxis”, and most patients (63%) were unsure if they are treated with those concentrates. FVIII concentrates with extended half-life, non-replacement, or gene therapy may even raise target trough levels (26). Gene therapy, non-replacement, or novel concentrates might be a future option, at least for some carefully selected patients (1, 27).

Less than half of the patients (43.6%) had received desmopressin in the past, and one-quarter of the patients (25.6%) used medications other than factor concentrates such as tranexamic acid or desmopressin as a current treatment option. Desmopressin is recommended for most patients with mild hemophilia A (28). The majority of the patients will reach adequate peak FVIII levels (≥30%) post-desmopressin and almost all if treatment decisions are adapted to desmopressin response testing results (16). However, FVIII levels >50% are considered safe for major surgery may not be reached (29). Desmopressin, recommended for patients with mild (and moderate) HA (1), was used by the patients in our survey for medical procedures, surgery, or accidents. Spontaneous or joint bleeding was less frequently treated with desmopressin, which is similar to the DYNAMO study. In the DYNAMO study, desmopressin was most commonly used for the treatment of minor wounds, oral cavity bleeds, and soft-tissue/(sub)cutaneous bleeds (16).

Approximately 60% of patients chose non-medicinal treatment or no medication for the treatment of bleeding episodes as compressions, a procedure applied by others for small bleeds or cuts, that is following the standard RICE (rest, ice, compression, and elevate) principle (30).

Half of the patients (50.0%) experienced complications due to untreated bleeding episodes in the past, which was lower than the percentage reported in the DYNAMO study (75%) (21). The treatment of mild hemophilia may be suboptimal regarding joint outcomes (25, 31). Increasing factor levels do not automatically correspond to a less severe phenotype (32); for individuals with HA, it is necessary to maintain factor levels of ≥15% (33) or > 20% (34) to prevent all spontaneous joint hemorrhages. Regression models of a longitudinal study (34) predicted 1.4 and 0.6 bleeds/year for patients with hemophilia A and B and factor levels of 15%, which seemed unlikely to prevent all joint bleeds. This may highlight the importance of adequate prophylaxis to avoid loss of joint range-of-motion and final hemophilic arthropathy after infrequent but ongoing bleeding episodes over time (35). Patients with mild HA may experience destruction of cartilage or mild-to-moderate synovitis (17). Repeated (limited or subclinical) or even single joint bleeding may lead to joint arthropathy, resulting in pain and decreased mobility of affected joints (1, 17, 18, 26). A large proportion of patients with non-severe hemophilia may therefore be at risk of long-term sequelae if not receiving more intensive treatment (31). Therefore, early detection of signs of joint damage (26), and patient education on prevention and early recognition of joint bleeding (21) are crucial (36).

Approximately, half of our patients (55.3%) visited the hemophilia center only when needed. Those patients requiring regular visits (44.7%) had to visit either once in every six months (52.9%) or only about once a year (41.2%). Patients with mild or mild-to-moderate hemophilia should visit a hemophilia treatment−/−comprehensive care center at least every two years (37). In daily routine, visits to hemophilia centers may be much more frequent with every 6 to 12 months, while the mean frequency could be even higher (19).

The generally (very) high satisfaction of the patients in our survey with the support from their hemophilia center and the treatment of bleeding episodes was also reported in another, yet small, survey from the United Kingdom, (30) and a larger study from the United States comprised patients with all severities (mild 32.8%), females patients with hemophilia, and patients with other bleeding disorders, such as von Willebrand disease (38).

In our survey, patients perceived hemophilia sometimes (43.2%) a burden during their daily life, and one-third (35.1%) of patients seldom had this feeling. This is slightly less than reported by other publications (26, 39). Mild hemophilia may negatively impact employment (19, 39) and have a moderate (59%) or large (53%) impact on education or work (39). Interestingly, the impact on education and work may be lower for patients with severe hemophilia, suggesting a better care reality for these patients (39). QoL data in mild hemophilia and comparisons to healthy controls are rare (26) indicating an unaddressed unmet need.

Most patients (73.0%) always/usually needed support from the hemophilia center, indicating the need for adequate treatment (10). However, patients may choose a wait-and-see approach before seeking healthcare services, depending on the severity of bleeding episodes and pain (30). Improved patient education may lead to earlier intervention and, thereby, timely treatment (7, 9).

Most patients had no problems in all five EQ-5D-5L dimensions (“mobility”, “self-care”, “usual activities”, “pain/discomfort”, and “anxiety/depression”), with the only exception of pain/discomfort. In this dimension, approximately similar proportions of patients experienced either no (41.2%) or a slight (47.1%) impairment. The patients experienced slight problems with mobility (21.6%) and usual activities (14.7%), and 26.5% of patients had slight symptoms of anxiety/depression. Our findings was similar to that of the B-Natural study HB cohort (31). The QoL was found to be affected in our study as well as in other studies (26). Patients with mild hemophilia may experience pain and long-term disabilities typically associated with severe hemophilia (40). Here, the percentage of slight/moderate pain/discomfort and no severe pain was lower than that in the B-HERO-S study, which included adult patients and caregivers of children with HB of all grades (19, 41). Patients with mild (and moderate) hemophilia reported higher acute and chronic pain levels than people without bleeding disorder, suggesting suboptimal treatment of joint disease (25), which again negatively impacts QoL (25, 31), strengthening the need for regular, close surveillance (17).

In this study, the median patient self-rated health, as reported by the EQ-VAS scale, was 80.0, ranging from 30 to 100, with a score of 100 equaling “best imaginable health”. This score was higher than that in the B-HERO-S study (41) and was similar to the P-FiQ study, which included both patients with HA and HB (19).

### 4.1 Limitations

As with any observational study, there is a risk of bias such as the selection bias due to differing patient populations visiting different centers. The patients who came to the centers in a certain period of time were approached, i.e., potentially those with a lower factor activity. It cannot be excluded that patients with more problems were more motivated to answer the questionnaire. The participation was lower (21.5 to 28.7%) than expected, which was also frequently observed in previous electronic/web-based surveys (14–18%) (42, 43). Increasing these through supportive measures, e.g., reminders, was not possible in our setting. Results may not be representative of all patients with mild hemophilia, as only those with smartphone/tablet access and the ability to scan QR codes could participate. Motivation/approaches in filling out questionnaires may differ, but retrospective verification is impossible due to anonymized data capture. Captured data were patient-reported and were not validated by a physician. Due to the survey design, reviewing unclear/incorrect information was impossible. While automated measures reduced the possibility of implausible data, it is important to note that missing/implausible data may still exist. The generation of irrelevant/double/fake datasets cannot be completely ruled out. Additionally, some data may be missing due to accidental or purposeful premature survey termination.

## 5 Conclusion

The definition “mild” based on the residual factor activity may not result in an actual perception of a mild disease by the affected patients. Mild hemophilia can be easily overlooked, leading to inadequate patient management, especially regarding joint problems/hemophilic arthropathy and lowering the patients’ QoL. Further research, such as longitudinal studies to track the progression of clinical outcomes or a study to determine whether individuals with higher factor activity have fewer emergency visits or scheduled appointments, might be useful in this patient population. Increased awareness of mild hemophilia is needed to improve diagnosis and treatment for these patients.

## Data availability statement

The original contributions presented in the study are included in the article/supplementary material, further inquiries can be directed to the corresponding author.

## Author contributions

RA: Conceptualization, Data curation, Funding acquisition, Investigation, Methodology, Project administration, Supervision, Validation, Writing – original draft, Writing – review & editing, Resources. GG: Resources, Writing – review & editing. SH: Resources, Writing – review & editing. KH: Resources, Writing – review & editing. CK: Investigation, Resources, Writing – review & editing. WM: Investigation, Resources, Writing – review & editing. CP: Resources, Supervision, Writing – review & editing. MO: Investigation, Supervision, Writing – review & editing.
